# Draft Genome Sequence of *Cyclobacterium qasimii* Strain M12-11B^T^, Isolated from Arctic Marine Sediment

**DOI:** 10.1128/genomeA.00642-13

**Published:** 2013-08-15

**Authors:** S. Shivaji, Sreenivas Ara, Aditya Singh, Anil Kumar Pinnaka

**Affiliations:** CSIR—Institute of Microbial Technology, Chandigarh, Indiaa; CSIR—Centre for Cellular and Molecular Biology, Hyderabad, Andhra Pradesh, Indiab

## Abstract

A 6.29-Mb genome sequence of *Cyclobacterium qasimii* strain M12-11B^T^, isolated from an Arctic marine sediment sample, is reported.

## GENOME ANNOUNCEMENT

*Cyclobacterium qasimii* strain M12-11B^T^ is a Gram-negative, nonmotile, horseshoe-shaped bacterium isolated from a marine sediment sample collected from Kongsfjorden, Svalbard, the Arctic. The 16S rRNA gene sequence similarity revealed the type strains of *Cyclobacterium amurskyense*, *Cyclobacterium marinum*, and *Cyclobacterium lianum* to be most closely related to *Cyclobacterium qasimii* M12-11B^T^, with similarity score values of 98.2, 96.8, and 93.3%, respectively (1).

The Roche 454 (FLX Titanium) pyrosequencing platform was used to perform whole-genome sequencing of *Cyclobacterium qasimii* M12-11B^T^. The sequencing yielded 136,911,603 bases in 320,561 reads, which is a 22× coverage of the genome. GS De Novo assembler (v 2.8) was used for the assembly of the raw sequencing data, which yielded a genome of 6,293,689 bp in 205 contigs with a G+C mol% of 42.11%. The smallest contig was 500 bp long and the largest contig 248,907 bp long. RAST (Rapid Annotation using Subsystem Technology) (2) was used to perform annotation of the assembly data, tRNA was predicted by tRNAscan-SE (v. 1.23) (3), and rRNA genes were predicted by RNAmmer (v. 1.2) (4).

The annotation of *Cyclobacterium qasimii* M12-11B^T^ indicated that there are a total of 5,958 predicted protein-coding regions and 45 RNA features. There are 86 genes providing resistance against antibiotics and toxic compounds, including genes for resistance against copper, cobalt, zinc, cadmium, mercury, and arsenic. We also found 197 genes involved in membrane transport. There were 121 stress response genes in the annotation of the genome, including 12 genes for heat shock, 4 genes for cold shock, 62 genes for oxidative stress, and 10 genes involved in detoxification.

The whole-genome sequencing of this bacterium isolated from the extremely cold climatic conditions of the Arctic region will help in the understanding of their adaptation and evolution to survive under these conditions.

### Nucleotide sequence accession numbers.

The draft genome sequence of *Cyclobacterium qasimii* strain M12-11B^T^ has been deposited in DDBJ/EMBL/GenBank under the accession number ATNM00000000. The version described in this paper is version ATNM01000000.
